# Alterations of peripheral blood lymphocyte subsets and plasma cytokines in Parkinson's disease and the parkinsonian subtype of multiple system atrophy

**DOI:** 10.3389/fnagi.2026.1990723

**Published:** 2026-10-28

**Authors:** Reyisha Taximaimaiti, Sheng Cai, Hongyan Li

**Affiliations:** 1 Department of Neurology, People's Hospital of Xinjiang Uygur Autonomous Region, Ürümqi, Xinjiang, China; 2 College of Life Sciences and Technology, Xinjiang University, Ürümqi, Xinjiang, China; 3 Zhejiang Province Key Laboratory of Anti-Cancer Drug Research, College of Pharmaceutical Sciences, Zhejiang University, Hangzhou, Zhejiang, China

**Keywords:** CD4–CD8– T cells, cytokines, lymphocyte subsets, multiple system atrophy, Parkinson's disease

## Abstract

**Objective:**

Peripheral immune system plays a crucial role in neurodegenerative diseases, but its alterations in Parkinson's disease (PD) and the parkinsonian subtype of multiple system atrophy (MSA-P) remained unclear. In this study, we investigated alterations of peripheral blood lymphocyte subsets and plasma cytokines among PD patients, MSA-P patients and healthy controls (HCs), and further compared their peripheral immune discrepancies.

**Methods:**

This observational study collected participants' peripheral venous blood from the Department of Neurology, People's Hospital of Xinjiang Uygur Autonomous Region from May 2025 to August 2026. The overall comparisons of lymphocyte subsets and cytokines among groups were conducted through MANCOVA, and distinguishing ability was tested through 5-fold cross-validated receiver operating characteristic curves and sensitivity analysis.

**Results:**

One hundred twenty PD patients, 45 MSA-P patients, and 119 HCs were enrolled in this study. MSA-P patients had younger age, younger age of symptom onset, shorter symptom duration, and lower drug dosage compared to PD patients. When PD patients and MSA-P patients had similar clinical performance, the count and percentage of CD4–CD8– T cells and IL-2 were significantly increased in MSA-P patients. And CD4–CD8– T cells exhibited a satisfactory discriminatory capacity to differentiate MSA-P from PD, which had no statistically significant correlation with clinical indicators.

**Discussion:**

Peripheral blood CD4–CD8– T cells might have the ability to differentiate MSA-P from PD, but relevant conclusions remained to be further verified.

## Introduction

Parkinson's disease (PD) and multiple system atrophy (MSA) are the most common α-synucleinopathies, both characterized by movement disorders, cognitive impairment and autonomic failure (Ndayisaba et al., 2025). Distinguishing the parkinsonian subtype of MSA (MSA-P) from PD at an early stage remains a major challenge in clinical practice, due to their overlapping clinical performance and similar pathological presentations. A spectrum of similar pathological alterations, including mitochondrial dysfunction, systemic chronic inflammation, disrupted iron metabolism, and impaired protein homeostasis, has been widely reported in both diseases (Lauritsen and Romero-Ramos, 2023; Chen et al., 2023) and discrepancies in pathological manifestations have also been gradually verified and illustrate the distinct mechanisms of these two diseases, such as the α-synuclein (α-syn) has different conformations and auto-antigenic responses (Ndayisaba et al., 2025). A growing number of approaches have been employed for the early identification of PD and MSA-P, among which altered peripheral immune system is receiving growing attention due to its many advantages such as convenient sample acquisition and high patient acceptability.

The peripheral immune system usually consists of circulating lymphocytes and monocyte in blood, immune cells localized in the enteric nervous system, and tissue-resident immune cells adjacent to peripheral autonomic ganglia (Castellani et al., 2023). Although inflammatory events in central nervous system (CNS) are the core driving factor in the pathogenesis of neurodegenerative diseases, alterations of peripheral immune have also been found to correlate and interact with CNS inflammatory responses (Moquin-Beaudry et al., 2025). This crosstalk is largely due to the widespread aberrant α-syn pathology simultaneously existing in both the central and the peripheral nervous system (Fonseca et al., 2026; Ndayisaba et al., 2025). Although the CNS is an immune-privileged organ and peripheral immune changes cannot fully recapitulate or precisely mirror the intrinsic pathological changes of CNS, peripheral blood can still provide valuable peripheral immune signatures reflecting disease-related pathological abnormalities in neurodegenerative diseases (Moquin-Beaudry et al., 2025). For example, T-helper 1 (Th1) and Th17 cells can recognize aberrant α-syn peptides in peripheral tissues and initiate targeted immune responses in blood, accompanied by elevated circulating pro-inflammatory cytokines such as interferon (IFN)-γ and tumor necrosis factor (TNF) (Lauritsen and Romero-Ramos, 2023). Circulating natural killer (NK) cells can exhibit cytotoxic activity changes and secrete IFN-γ, and circulating B cells can differentiate into plasma cells and generate auto-antibodies targeting α-syn (Roodveldt et al., 2024). Furthermore, targeted inhibition of excessive peripheral adaptive immune responses and subsequent immune cell infiltration has been proven to improve the efficacy of some new therapies, such as cell replacement and stem-cell therapy, thereby providing a potential approach to alleviate clinical deterioration in neurodegenerative diseases patients (Park et al., 2023). Collectively, as a highly accessible and minimally invasive sampling source, peripheral immune system alterations may offer us an opportunity to explore potential biomarkers to early differential diagnosis among neurodegenerative diseases including MSA-P and PD.

Peripheral blood lymphocyte subsets and cytokines analysis are among the most widely used approaches for characterizing peripheral immune profiles, and has been validated to have a satisfactory capacity in identifying peripheral immunity alterations in neurodegenerative diseases (Yuan et al., 2024). For example, PD patients exhibit elevated activity of CD4+ T cells (including Th1 and Th17), CD8+ T cells, and B cells, along with decreased counts of naive CD4+ and CD8+ T cells; as well as elevated pro-inflammatory cytokines including interleukin (IL)-1β, IL-2, IL-4, IL-6, IL-10, IL-17, IFN-γ and TNF compared to healthy controls (HCs) (Iyengar et al., 2025; Lauritsen and Romero-Ramos, 2023; Yan et al., 2021). In MSA patients, increased monocyte counts and decreased naive CD4+ T cell have been observed, whereas both increase and decrease of CD56+ NK cells have been reported (Moquin-Beaudry et al., 2025; Jensen et al., 2021; Yuan et al., 2024). And in isolated rapid eye movement sleep behavior disorder (iRBD), a typical prodromal symptom of PD, patients have been observed to have reduced counts of total lymphocyte, CD3+ T cells, and anti-inflammatory Th2 cells; as well as elevated pro-inflammatory Th1 cells and IFN-γ + CD8 + T cells, which are positively correlated with the severity of autonomic dysfunction and psychiatric complications (Zheng et al., 2024; He et al., 2022). Overall, these findings indicate that peripheral blood lymphocyte subsets and cytokines are significantly alternated in neurodegenerative diseases, which may offer us an opportunity to search for appropriate immune biomarkers to distinguish MSA-P from PD.

In this study, we investigated alterations of peripheral blood lymphocyte subsets and plasma cytokines among PD patients, MSA-P patients and HCs, and further compared their peripheral immune discrepancies. We aimed to identify potential immune biomarkers to distinguish MSA-P from PD.

## Methods

### Participants enrollment

One hundred twenty PD patients, 45 MSA-P patients, and 119 HCs were enrolled in this study from Department of Neurology, People's Hospital of Xinjiang Uygur Autonomous Region from May 2025 to August 2026 (Figure 1). The diagnosis of idiopathic PD and MSA-P was confirmed by two senior neurologists independently according to validated clinical diagnostic criteria (Postuma et al., 2015; Gregor et al., 2022). MSA-P patients were classified based on the main symptoms of Parkinsonism at initial diagnosis.

**Figure 1:**
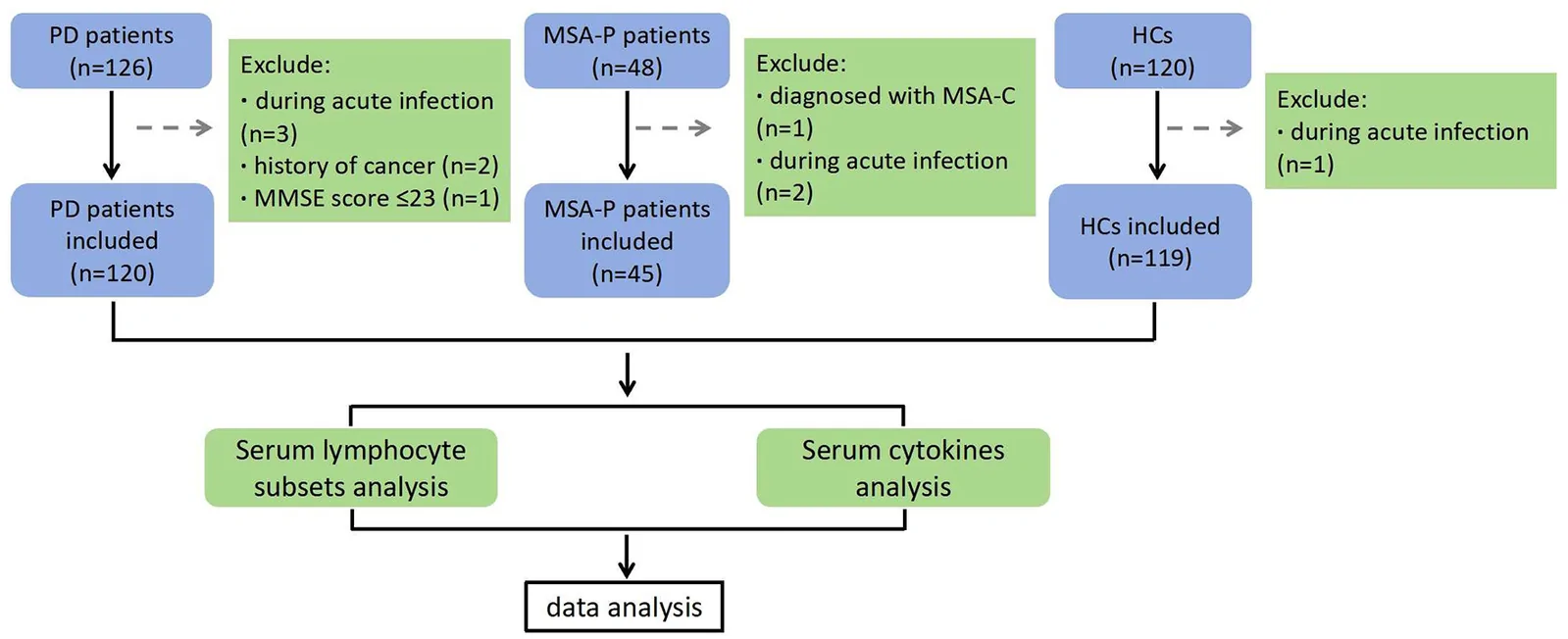
Flowchart of Inclusion and Exclusion. PD, Parkinson's disease; MSA-P, the Parkinsonian subtype of multiple system atrophy; HC, health control; MMSE, Mini-Mental State Examination, MSA-C, the cerebellar subtype of multiple system atrophy.

Inclusion criteria for PD patients and MSA-P patients included: (1) 40–85 years of age; (2) no history of neurological diseases (other than PD and MSA), or severe mental illness; (3) no history of cancer; (4) no history of autoimmune diseases or use of immunomodulatory drugs; (5) not during acute infection period at the time of testing, and no history of chronic infections (e.g., human immunodeficiency virus [HIV], hepatitis B virus [HBV], and syphilis, etc.); (6) not in the pregnancy period; (7) no use of antibiotic within 2 months; (8) no vaccination within 2 months; (9) Mini-Mental State Examination (MMSE) score >23. Inclusion criteria for HCs included: (1) no history/family history of movement disorders; (2) other criteria were unified with PD patients and MSA-P patients.

### Ethics statement

The Ethics Committee of People's Hospital of Xinjiang Uygur Autonomous Region approved the protocol (KY2025022107). Written informed consent was obtained from all participants or their legal guardians. All processes of this study were carried out in accordance with the Declaration of Helsinki.

### Data collection

Demographic information, including sex, age, years of education, body mass index (BMI), MMSE, Montreal Cognitive Assessment (MoCA), Activity of Daily Living Scale (ADL), Hamilton Depression Scale (HAMD), and Hamilton Anxiety Scale (HAMA), was collected from all participants. Clinical information, including, age of symptom onset, symptom duration, Unified Parkinson's Disease Rating Scale-part III (UPDRS-III) score, Hoehn-Yahr score, levodopa equivalent daily dose (LEDD), was collected from PD patients and MSA-P patients according to medical history records and patients' reconfirmation.

Peripheral blood lymphocyte subsets analysis and plasma cytokines analysis were performed at the Laboratory Department of People's Hospital of Xinjiang Uygur Autonomous Region. The procedure was as follows: after an 8-h overnight fast, trained nurses collected 15 mL of peripheral venous blood before 7:00 a. m. into EDTA-anticoagulant tubes. Peripheral blood lymphocyte subsets analysis was conducted within 2 h using the EasyCell 206A1 system (Wellgrow Biotechnology, China) in accordance with the manufacturer's instructions. Briefly, after mixing the blood samples thoroughly, added 1 mmol/L EDTA. Twenty microliter of BD Multitest 6-Color (CD3/CD4/CD8/CD16+CD56/CD19/CD45) TBNK (BD Biosciences, United States) was added into 50 μL of the blood sample, mixed well, and incubated at room temperature for 15 min. Then 450 μL of BD FACS Lysing Solution (BD Biosciences, USA) was added, mixed well, and incubated at room temperature for 15 min before the flow cytometer. The gating strategy for flow cytometry was shown in Figure 2. Identified lymphocytes from WBC (CD45+) firstly, then identified T cells (CD3+), B cells (CD19+), NK cells (CD16+CD56+), and NKT cells (CD3+CD16+CD56+) sequentially. T cells were further subdivided into CD4+ T cells (CD4+), CD8+ T cells (CD8+), CD4+CD8+ T cells (CD4+CD8+), and CD4–CD8– T cells (CD4–CD8–) separately. Percentages of each lymphocyte subset were calculated with the count of total lymphocytes as the denominator. The EasyCell 206A1 system automatically calculated cell counts and corresponding percentages. Fluorophore compensation was performed using single-color bead controls. All gating was manually checked, and gating quality control was routinely performed to remove cell debris and doublets. Plasma was separated from whole blood by centrifugation at 1,000 × g for 15 min at 4 °C and cytokines analysis was conducted within 2 h using the Human Inflammatory Cytokine Kits (BD Biosciences, United States) in accordance with the manufacturer's instructions. Briefly, 50 μl of test sample or standard dilutions was added to assay tubes containing 50 μl pre-treated Capture Beads, and incubated at room temperature in the dark for 1.5 h. Added 1ml of wash buffer in each tube, and centrifuged at 200 × g for 5 min. After discarding the supernatant, added 50 μl of the PE detection reagent to each tube, and incubated at room temperature in the dark for 1.5 h. Added 1ml of wash buffer to each tube, centrifuged at 200 × g for 5 min, discarded the supernatant, and added 300μl of wash buffer to resuspend the bead pellet. Then the EasyCell 206A1 system was used to analysis the concentration of interleukin (IL)-1β, IL-2, IL-4, IL-6, IL-10, IL-12p70, IL-17, IFN-γ, and TNF-α.

**Figure 2:**
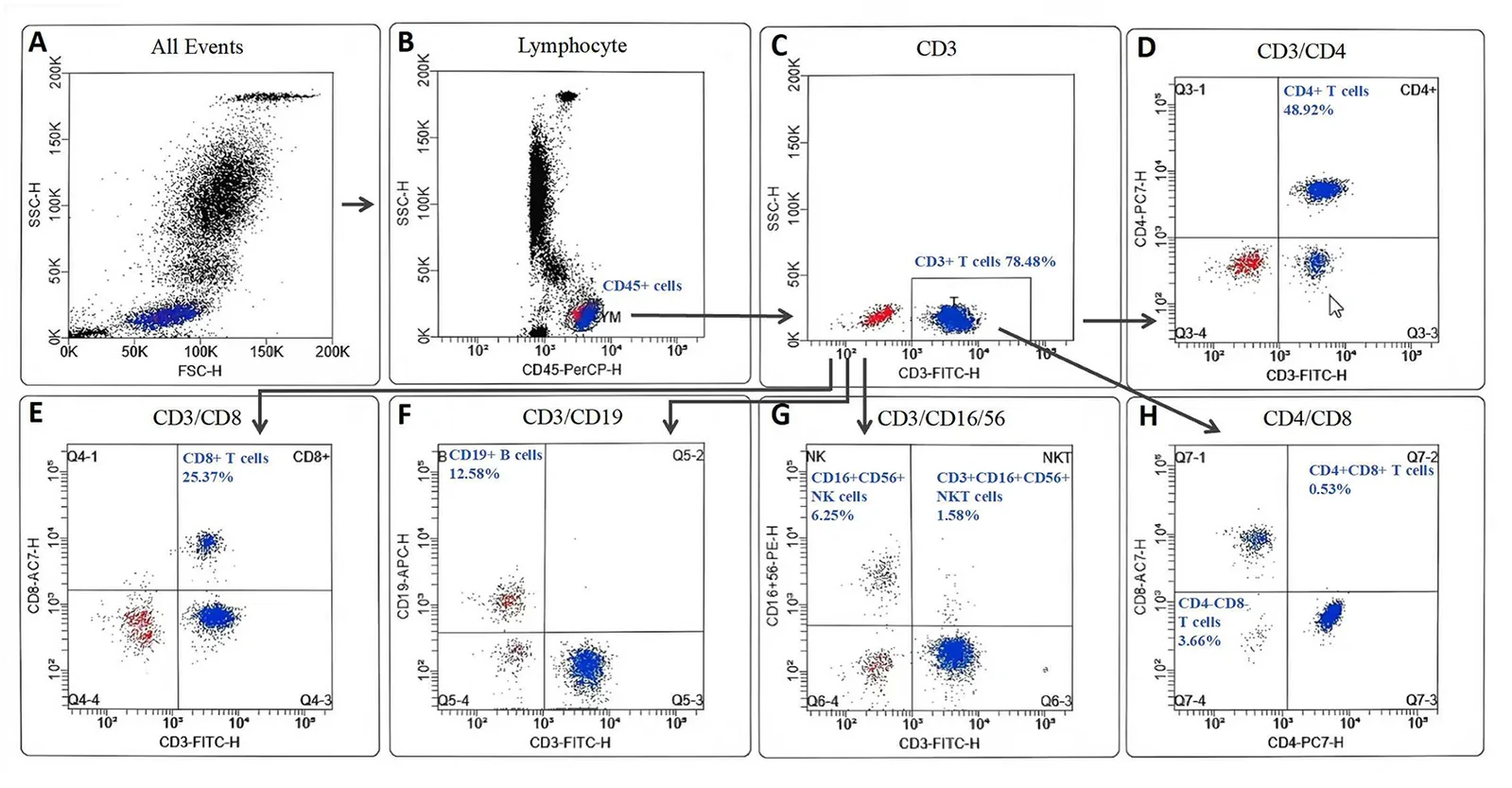
The gating strategy of flow cytometry for peripheral blood lymphocyte subsets analysis. **(A, B)** identify lymphocytes from white blood cells (CD45+); **(C)** identify t cells (CD45+CD3+) from lymphocytes; **(D)** identify CD4+ T cells (CD45+CD3+CD4+); **(E)** identify CD8+ T cells (CD45+CD3+CD8+); **(F)** identify B cells (CD45+CD3-CD19+); **(G)** identify NK cells (CD45+CD3-CD16+CD56+) and NKT cells (CD45+CD3+CD16+CD56+); **(H)** identify CD4+CD8+ T cells (CD45+CD3+CD4+CD8+) and CD4-CD8- T cells (CD45+CD3+CD4-CD8–). **(A–C)** CD45+CD3+ lymphocytes were set as blue, and CD45+CD3– lymphocytes were set as red; **(D–G)** red/blue was set to distinguish CD3–/CD3+. When calculating percentages of each lymphocyte subset, the count of total lymphocytes was set as the denominator. These flow cytometry plots were from a PD patient. Representative plots for MSA-P patients and HCs are showed in Supplementary Figure 1.

### Sample size calculation

G^*^Power v3.1.9.7 was used to calculate the sample size. Selecting the “F tests,” “MANOVA: Global effects,” and “A priori: Compute required sample size”; giving the effect size f2 at 0.2, α error probability at 0.05, the power at 0.80, number of groups was 3, response variable was 1 (have PD or MSA-P); and obtained a total sample size of 54. As three pairwise comparisons were required for three groups (PD patients vs. HCs, MSA-P patients vs. HCs, and PD patients vs. MSA-P patients), the *post-hoc* Bonferroni adjusted α value was 0.05/3 ≈ 0.017, obtained a total sample size of 69. With 20% covariate influence and 20% sample attrition, at least 100 participants in total and 34 participants each group were required to satisfy the actual power of the sample. This study met the sample size requirements.

### Statistical analysis

FlowJo v10.8 (BD Biosciences, United States) was used for flow cytometry data analysis, and R v4.5.1 (Posit Software, United States) was used for statistical analysis. Origin 2024 (OriginLab, United States), GraphPad Prism v10.1.2 (GraphPad Software, United States), and R v4.5.1 were used for statistical drawing. Quantitative variables were presented as mean ± standard deviation (SD), and were checked for data normality and homogeneity of variance via the Shapiro–Wilk test and the Levene's test. Categorical variables were presented as frequencies and percentages (%), and compared using the χ^2^ test.

In demographic and clinical features part, one-way ANOVA was used for overall comparisons across three groups, followed by *post-hoc* Bonferroni comparisons between groups. For clinical quantitative indicators only for PD patients and MSA-P patients, two-tailed independent samples *t*-tests were adopted. In lymphocyte subsets analysis and cytokines analysis part, the Shapiro–Wilk test and the Levene's test were used to evaluate data normality and homogeneity of variance. One-way MANCOVA (adjusts for groups, sex, age, MMSE, MoCA, HAMD, and HAMA) was used to calculate overall comparison F, P, and Partial η2. *Post-hoc* Bonferroni was used to calculate between-group comparison p, and the Benjamini–Hochberg method was used to adjust *p*-value to the false discovery rate (FDR) when appropriate. Binary Logistic analysis (adjusted for sex, age, symptom duration, UPDRS-III score, MMSE, MoCA, LEDD, HAMD, and HAMA) was used to explore the relationship between peripheral blood lymphocyte subsets/plasma cytokines and the probability of MSA-P. And variance inflation factor (VIF) analysis and Bootstrap resampling (1,000 replicates) were used to evaluate the stability of the multivariable logistic regression models. Partial Pearson correlation analysis was used to evaluate correlations between clinical indicators and peripheral blood lymphocyte subsets/plasma cytokines in PD patients and MSA-P patients. Receiver operating characteristic (ROC) curve analysis was conducted to evaluate the discriminatory ability of peripheral blood lymphocyte subsets/plasma cytokines to differentiate MSA-P from PD. For variables showing statistically significant differences, a 5-fold cross-validation (repeated 100 times) was performed for internal validation, and the area under curves (AUC) with corresponding 95% confidence intervals (CIs) were calculated. Decision curve analysis (DCA) was performed to quantify the net benefit of the prediction model across varying threshold probabilities and assess its practical decision-making utility. Calibration curves based on bootstrap resampling (*B* = 500) were constructed to evaluate the agreement between model-predicted probabilities and observed probabilities. Meanwhile, considering standard 5-fold cross-validation had data leakage and thereby overestimate model performance, we additionally conducted repeated nested stratified 5-fold cross-validation to obtain an unbiased estimate of model generalization. Next, stratified models were established for PD patients and MSA-P patients based on sex, UPDRS-III score, and LEDD. Sensitivity analysis was then carried out to verify the discriminatory power of peripheral blood lymphocyte subsets. The optimal cut-off values for UPDRS-III score and LEDD were determined via ROC curve analysis by maximizing the Youden index (J = Sensitivity + Specificity – 1). Then, residual-based inverse probability weighting (IPW) (adjusted for sex, age, symptom duration, UPDRS-III score, MMSE, MoCA, LEDD, HAMD, and HAMA) was applied to mitigate confounding. Covariate balance was evaluated by Pearson correlation coefficients between covariates and exposure, with adequate balance defined as |*r*| < 0.1. A two-tailed *P*-value < 0.05/FDR < 0.05 indicated statistically significant.

## Result

### MSA-P patients were younger yet exhibited similar clinical profiles to PD patients

One hundred twenty PD patients, 45 MSA-P patients, and 119 HCs were enrolled in this study (Figure 1), and completed peripheral blood lymphocyte subsets analysis (Figure 2 and Supplementary Figure 1) and plasma cytokines analysis. As presented in Table 1, three groups had similar sex, years of education and BMI. PD patients had the oldest age, whereas MSA-P patients were the youngest. Both PD patients and MSA-P patients performed worse on MMSE, MoCA, ADL, HAMD, HAMA compared to HCs, and PD patients had higher HAMD and HAMA scores than MSA-P patients. PD patients and MSA-P patients showed similar UPDRS-III score and Hoehn-Yahr stages, while PD patients had older age of symptom onset, longer symptom duration, and higher LEDD compared to MSA-P patients.

**Table 1:** Demographic and clinical features of PD patients, MSA-P patients, and HCs.

Variables	PD patients (*n* = 120)	MSA-P patients (*n* = 45)	HCs (*n* = 119)	Overall comparison *P*	Between-group comparison *P*		
					PD vs. HCs	MSA-P vs. HCs	PD vs. MSA-P
Sex (male/female)	51/69	18/27	47/72	0.89	–	–	–
Age (years)	68.0 ± 9.3	57.2 ± 9.4	62.3 ± 9.5	**< 0.001**	**< 0.001**	**< 0.01**	**< 0.001**
Years of education (years)	9.6 ± 4.3	10.9 ± 3.3	10.6 ± 3.9	0.08	0.18	1.00	0.20
BMI (kg/m^2^)	25.1 ± 3.3	24.8 ± 4.3	25.5 ± 3.7	0.48	1.00	0.79	1.00
MMSE	26.6 ± 1.9	26.0 ± 1.8	28.5 ± 1.6	**< 0.001**	**< 0.001**	**< 0.001**	0.16
MoCA	22.5 ± 3.3	21.5 ± 2.9	25.9 ± 3.0	**< 0.001**	**< 0.001**	**< 0.001**	0.21
ADL	29.3 ± 11.7	31.6 ± 10.2	20.5 ± 1.0	**< 0.001**	**< 0.001**	**< 0.001**	0.35
HAMD	28.3 ± 6.0	25.6 ± 4.7	22.7 ± 2.7	**< 0.001**	**< 0.001**	**< 0.01**	**< 0.01**
HAMA	22.0 ± 5.8	19.6 ± 5.1	17.4 ± 3.3	**< 0.001**	**< 0.001**	**0.03**	**0.01**
Age of symptom onset (years)	64.0 ± 9.8	54.7 ± 9.7	–	–	–	–	**< 0.001**
Symptom duration (years)	4.0 ± 3.5	2.5 ± 2.1	–	–	–	–	**0.001**
UPDRS-III score	19.5 ± 8.6	20.6 ± 7.8	–	–	–	–	0.44
Hoehn-Yahr (1/2/3/4)	30/40/39/11	6/15/22/2	–	–	–	–	0.15
LEDD (mg/day)	367.2 ± 288.3	164.6 ± 235.3	–	–	–	–	**< 0.001**

PD, Parkinson's disease; MSA-P, the Parkinsonian subtype of multiple system atrophy; HC; health control; BMI; body mass index; MMSE, Mini-Mental State Examination; MoCA, Montreal Cognitive Assessment; ADL, Activity of Daily Living Scale; HAMD, Hamilton Depression Scale; HAMA, Hamilton Anxiety Scale, UPDRS-III, Unified Parkinson's Disease Rating Scale-part III; LEDD, levodopa equivalent daily dose.

One-way ANOVA was used to calculate overall comparison P, and post-hoc Bonferroni was used to calculate between-group comparison *P*.

Bold denotes *p* < 0.05.

### CD4–CD8– T cells were significantly increased in MSA-P patients and exhibited a satisfactory discriminatory power to differentiate MSA-P from PD

Figure 3 and Supplementary Tables 1, 3 listed the results of peripheral blood lymphocyte subsets analysis and plasma cytokines analysis. The count and percentage of CD4–CD8– T cells [MSA-P vs. HCs: FDR < 0.001; MSA-P vs. PD: FDR = 0.02 (count)/FDR < 0.01 (percentage)], and IL-2 (FDR < 0.01) were significantly increased in MSA-P patients compared to PD patients and HCs. PD patients showed higher count and percentage of CD4–CD8– T cells (FDR < 0.001) and CD3+CD16+CD56+ NKT cells [FDR = 0.02 (count)/FDR < 0.01 (percentage)], and TNF-α (FDR = 0.01) compared to HCs. The main effect tests (Supplementary Tables 2, 4) indicated that group effects (PD/MSA-P/HCs) were the main reason for these differences.

**Figure 3:**
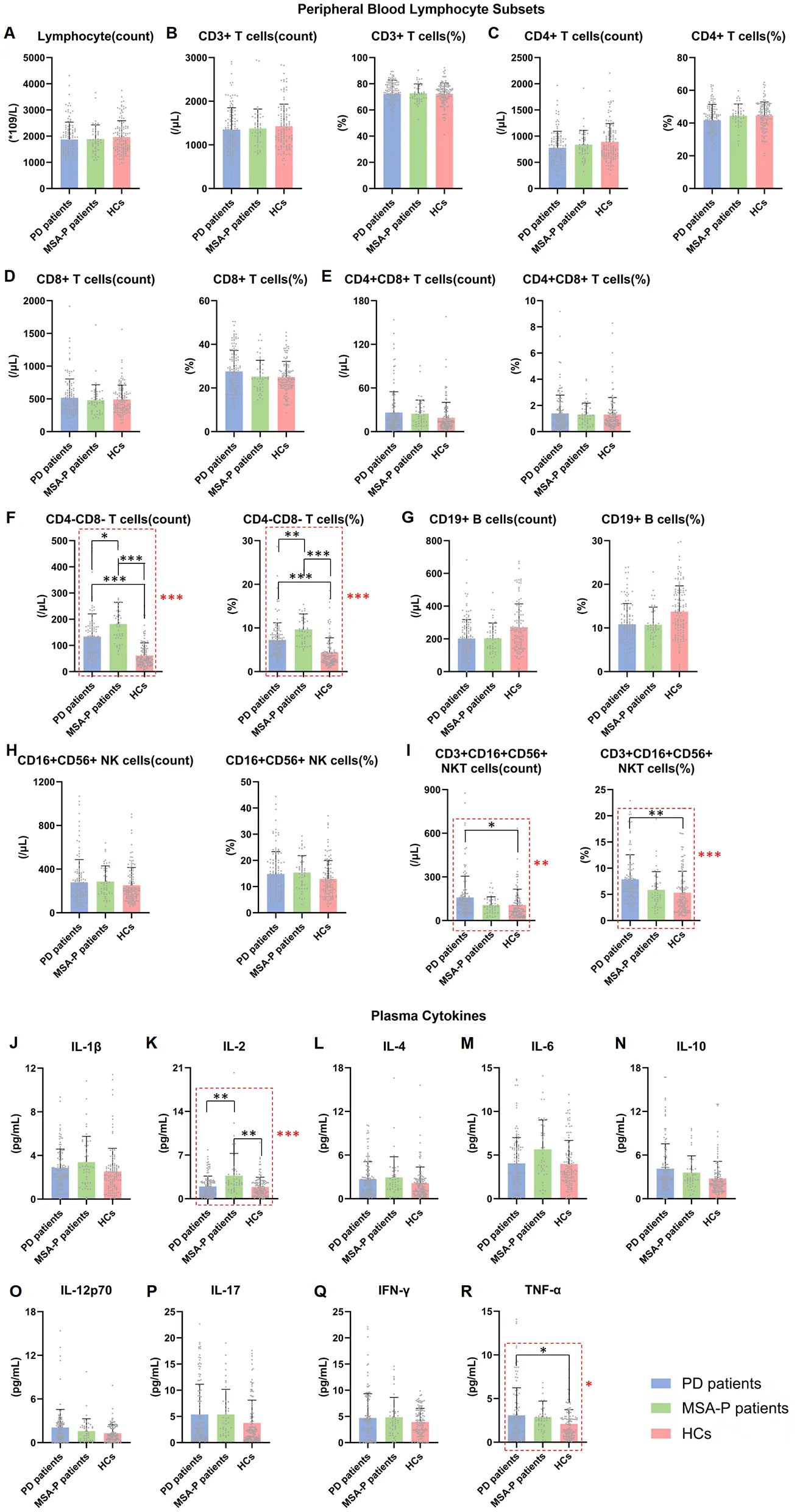
Peripheral blood lymphocyte subsets and plasma cytokines in PD patients, MSA-P patients, and HCs. **(A)** The count of total peripheral blood lymphocytes; the count and percentage (%) of CD3+ T cells **(B)**, CD4+ T cells **(C)**, CD8+ T cells **(D)**, CD4+CD8+ T cells **(E)**, CD4–CD8– T cells **(F)**, CD19+ B cells **(G)**, CD16+CD56+ NK cells **(H)**, CD3+CD16+CD56+ NKT cells **(I)** in peripheral blood. The concentration of IL-1β **(J)**, IL-2 **(K)**, IL-4 **(L)**, IL-6 **(M)**, IL-10 **(N)**, IL-12p70 **(O)**, IL-17 **(P)**, IFN-γ **(Q)**, and TNF-α **(R)** in peripheral blood. PD, Parkinson's disease; MSA-P, the Parkinsonian subtype of multiple system atrophy; HC, health control; NK cells, natural killer cells; NKT cells, natural killer T cells; IL, interleukin; IFN, interferon; TNF, tumor necrosis factor. PD patients, *n* = 120; MSA-P patients, *n* = 45; HCs, *n* = 119. MANCOVA (adjusts for groups, sex, age, MMSE, MoCA, HAMD, and HAMA) was used to calculate overall comparison P, and the Benjamini–Hochberg method was used to adjust between-group comparison p to the FDR. The red dashed box marks statistically significant differences in overall comparisons based on MANCOVA (Supplementary Tables 1, 3), with group effects were the main reason for these differences (Supplementary Tables 2, 4). Box height indicates the mean value; line segments with horizontal end caps represent the standard deviation. * denotes statistically significant differences between groups. *, P/FDR < 0.05; **, P/FDR < 0.01; ***, P/FDR < 0.001. For details, please refer to Supplementary Tables 1, 3.

The results of Logistic analysis (Supplementary Table 5) indicated that the count and percentage of CD4–CD8– T cells and CD3+CD16+CD56+ NKT cells, IL-2, and IL-6 had potentially distinguishing ability. VIF analysis indicated no severe multicollinearity (all VIF < 10), and Bootstrap resampling confirmed that the associations remained stable for the count and percentage of CD4–CD8– T cells, the percentage of CD3+CD16+CD56+ NKT cells, IL-2, and IL-6. (Supplementary Table 6) Partial Pearson correlation analysis (Figure 4) observed no common correlation with statistically significant between clinical indicators and peripheral blood lymphocyte subsets/plasma cytokines in PD patients and MSA-P patients.

**Figure 4:**
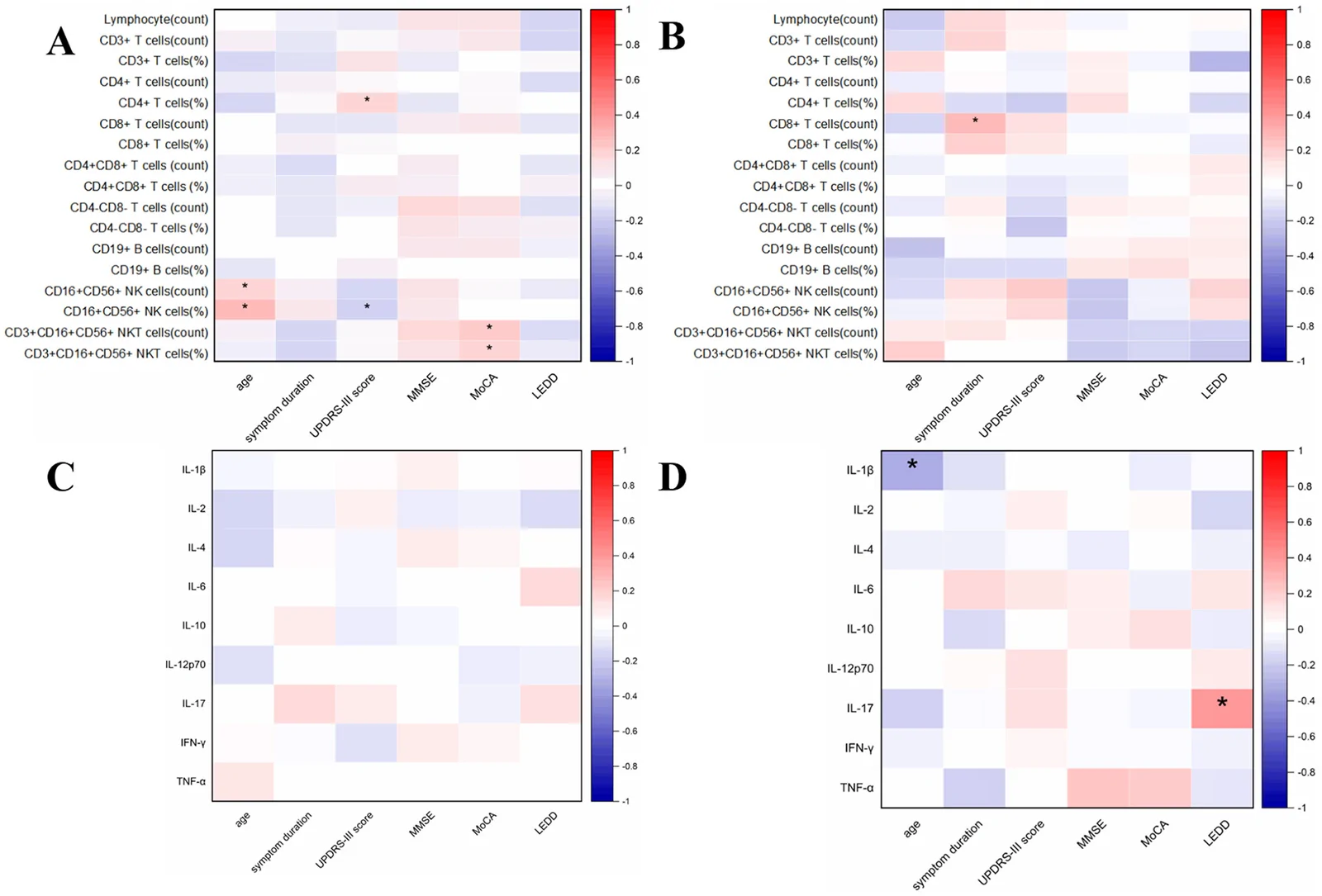
Heatmap of partial pearson correlation coefficient of peripheral blood lymphocyte subsets and plasma cytokines in PD patients and MSA-P patients. PD, Parkinson's disease; MSA-P, the Parkinsonian subtype of multiple system atrophy, UPDRS-III, Unified Parkinson's Disease Rating Scale-part III; MMSE, Mini-Mental State Examination; MoCA, Montreal Cognitive Assessment; LEDD, levodopa equivalent daily dose; NK cells, natural killer cells; NKT cells, natural killer T cells; IL, interleukin; IFN, interferon; TNF, tumor necrosis factor. **(A)** Correlation between peripheral blood lymphocyte subsets and clinical features in PD patients; **(B)** correlation between peripheral blood lymphocyte subsets and clinical features in MSA-P patients; **(C)** correlation between plasma cytokines and clinical features in PD patients; **(D)** correlation between plasma cytokines and clinical features in MSA-P patients.

Five-fold cross-validated ROC curves (Figures 5A, B) indicated that the count and percentage of CD4–CD8– T cells, the percentage of CD3+CD16+CD56+ NKT cells, IL-2, and IL-6 might possess discriminatory ability for distinguishing MSA-P patients from PD patients. Notably, the AUC for the count and percentage of CD4–CD8– T cells exceeded 0.70, suggesting satisfactory discrimination performance. We further established a model with variables in the 5-fold cross-validated ROC curves to draw the DCA curve (Figure 5C) and the bootstrap calibration curves (Figure 5D). The DCA curve suggested good clinical applicability and favorable net positive benefit, and the bootstrap calibration curves demonstrated a reliable discriminatory accuracy between predicted and actual probabilities (mean absolute error = 0.03, mean squared error = 0.00143, 0.9 quantile of absolute error = 0.063). Nested cross-validation (Figure 5E) was applied to evaluate the generalization performance of model constructed using variables identified in the 5-fold cross-validated ROC analysis. The result showed that the model achieved good discriminative ability for distinguishing PD from MSA-P patients, with a mean AUC of 0.90 (95% CI: 0.85–0.98).

**Figure 5:**
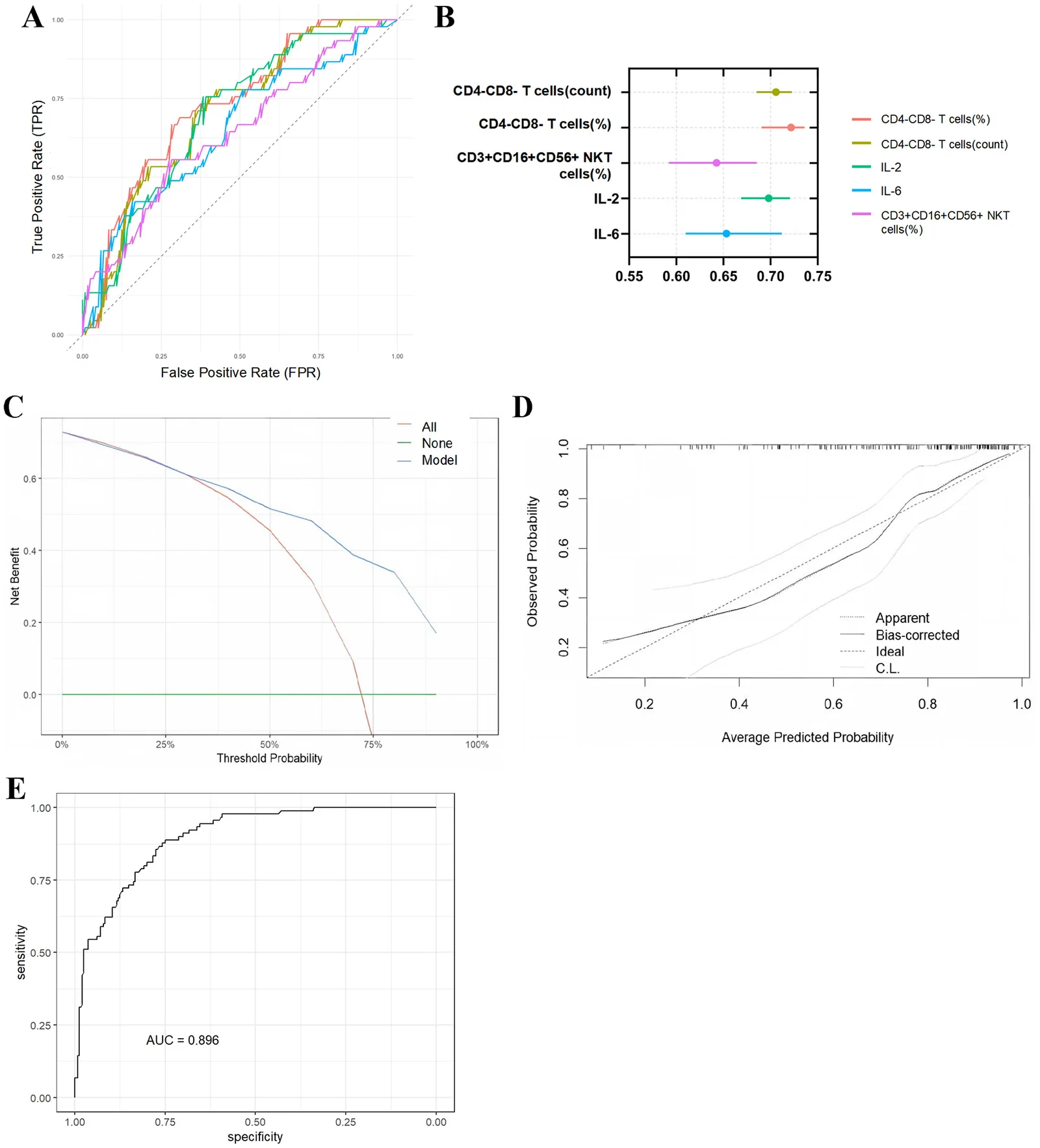
5-Fold Cross-validated ROC Curves, DCA Curve, and Calibration Curve of Peripheral Blood Lymphocyte Subsets and Plasma Cytokines in PD Patients and MSA-P Patients. ROC: receiver operating characteristic, DCA: decision curve analysis, PD, Parkinson's disease, MSA-P, the Parkinsonian subtype of multiple system atrophy; IL, interleukin; NKT cells, natural killer T cells. PD patients, *n* = 120; MSA-P patients. **(A)** 5-fold cross-validated ROC curves of peripheral blood lymphocyte subsets and plasma cytokines with statistically significant results between PD patients and MSA-P patients; **(B)** corresponding AUC and 95%CI in 5-fold cross-validated ROC curves [IL-2: 0.70(0.67–0.72); IL-6: 0.65(0.61–0.71); CD4–CD8– T cells(count):0.71(0.69–0.72); CD4–CD8– T cells(%): 0.72(0.69–0.74); CD3+CD16+CD56+ NKT cells(%): 0.64(0.59–0.69)]; **(C)** DCA curve for 5-fold cross-validated ROC curves (set peripheral blood lymphocyte subsets and plasma cytokines in 5-fold cross-validated ROC curves as a Model); **(D)** bootstrap calibration curve for Model of 5-fold cross-validated ROC curves (mean absolute error = 0.03, mean squared error = 0.00143, 0.9 quantile of absolute error = 0.063); **(E)** Nested cross-validation ROC curve (mean AUC = 0.90, 95% CI: 0.85–0.98).

### Sensitivity analysis: CD4–CD8– T cells maintained discriminatory ability to distinguish MSA-P from PD across different models

To verify whether CD4–CD8– T cells could distinguish MSA-P patients from PD patients across different patient subgroups, we performed a series of sensitivity analysis. Supplementary Table 7 listed the data profiles of participants included in the sensitivity analysis, and Table 2 listed the corresponding results.

**Table 2:** Sensitivity analysis for the count of peripheral blood CD4–CD8– T cells in PD patients and MSA-P patients.

Models		*n* (PD/MSA-P)	Unadjusted		IPW–adjusted	
			P	OR (95% CI)	P	OR (95% CI)
Model 1: sex	Male	51/18	**0.02**	1.40 (1.21–1.52)	**0.01**	1.43 (1.18–1.66)
	Female	69/27	**< 0.01**	1.48 (1.25-1.74)	**< 0.001**	1.78(1.15–2.75)
Model 2: UPDRS-III score	>20	56/22	**0.001**	1.56 (1.24–1.97)	**< 0.001**	1.63 (1.17–2.27)
	≤20	64/23	**0.01**	1.45 (1.21–1.68)	**< 0.01**	1.47 (1.18–1.75)
Model 3: LEDD	>275	72/13	**0.01**	1.48 (1.12–1.70)	**< 0.01**	1.55 (1.07–1.91)
	≤275	48/32	**< 0.01**	1.49 (1.27–1.75)	**< 0.01**	1.51 (1.16–1.97)

PD, Parkinson's disease; MSA-P, the Parkinsonian subtype of multiple system atrophy; UPDRS-III, Unified Parkinson's Disease Rating Scale-part III; LEDD, levodopa equivalent daily dose; IPW, inverse probability weighting; OR, odds ratio; CI, confidence interval.

Model 1: adjusted for age, symptom duration, UPDRS-III score, MMSE, MoCA, LEDD, HAMD, and HAMA.

Model 2: adjusted for sex, age, symptom duration, MMSE, MoCA, LEDD, HAMD, and HAMA.

Model 3 adjusted for sex, age, symptom duration, UPDRS-III score, MMSE, MoCA, HAMD, and HAMA.

OR values correspond to the count of CD4–CD8– T cells.

Bold denotes *p* < 0.05.

Three models (Model 1–3) were established according to different sex, UPDRS-III score and LEDD, and participants were stratified accordingly. We assessed the discrimination performance of the count of CD4–CD8– T cells in different models respectively to evaluate the consistency of their discriminatory ability to distinguish MSA-P patients from PD patients across different models. Sensitivity analysis for Model 1–3 all suggested satisfactory discriminatory power of the count of CD4–CD8– T cells for potentially differentiating MSA-P from PD. Covariate balance assessment (Supplementary Table 8) confirmed acceptable covariate balance after residual-based weighting. It was worth mentioning that this stratified analysis was exploratory owing to the limited sample size, further validation in larger cohorts was required.

## Discussion

Several main findings had arisen from this study: (1) When PD patients and MSA-P patients had similar clinical performance, MSA-P patients had younger age, younger age of symptom onset, shorter symptom duration, and lower drug dosage. (2) The count and percentage of CD4–CD8– T cells and IL-2 were significantly increased in MSA-P patients compared to PD patients and HCs; and the count and percentage of CD4–CD8– T cells and CD3+CD16+CD56+ NKT cells, and TNF-α were significantly increased in PD patients compared to HCs. (3) When PD patients and MSA-P patients had similar clinical performance, CD4–CD8– T cells were significantly increased in MSA-P patients, and exhibited a satisfactory discriminatory capacity to differentiate MSA-P from PD, which had no statistically significant correlation with clinical indicators.

In our study, the count and percentage of CD4–CD8– T cells were significantly increased in MSA-P patients compared to PD patients and HCs, and could maintain discriminatory ability to distinguish MSA-P from PD across different patient subgroups. The CD4–CD8– T cells, also called double negative (DN) T cells, account for 1–5% in circulating blood in healthy population, whose active form usually classified as cytotoxic and pro-inflammatory lymphocyte subset (Wu et al., 2022) CD4–CD8– T cells mainly originate from both thymus and periphery, they express CD3 but lack the CD4 and CD8 co-receptors due to gene expression abnormality or certain defects in signal transduction pathway (Velikkakam et al., 2022). CD4–CD8– T cells lack well-defined cell markers, which makes it difficult to characterize them into different subphenotypes and thus exclusion criteria are often used instead (Li et al., 2025b). Depending on the environment, CD4–CD8– T cells can exhibit pro- or anti-inflammatory function, whose activated form can stably secrete a series of cytokines including IL-2, IL-10, IL-17, IFN-γ and TNF-α (Whyte et al., 2022; Wu et al., 2022). In previous studies, activated CD4–CD8– T cells have been detected existing in circulating blood and cerebrospinal fluid of PD patients, and are capable of crossing the blood-brain barrier and infiltrating the CNS (Wang et al., 2021). In MSA patients, although systemic immune activation has been observed, studies on the distribution and function of peripheral blood CD4–CD8– T cells are still lacking (Moon et al., 2026). In our study, PD patients and MSA-P patients both showed an abnormally elevated trend of CD4–CD8– T cells, which might be an important part of the adaptive immune response to α-syn. However, considering TCR αβ*/*γδ phenotyping was not performed in this study, and cell status and specific function had not been evaluated, further sub-classification of lymphocytes is recommended to explore the mechanism underlying this phenomenon. In addition, count and percentage of CD3+CD16+CD56+ NKT cells were significantly increased in PD patients compared to HCs, whereas no significant differences were observed compared to MSA-P patients. This observational finding was still preliminary and exploratory, and therefore required further verification in larger cohorts and mechanism research.

IL-2 was also elevated in MSA-P patients compared to PD patients and HCs in our study. IL-2 is a key homeostatic cytokine balancing the pro-inflammatory and anti-inflammatory signals, equilibrium the activity of regulatory T cells (Tregs) and pro-inflammatory T cells (e.g. Th1 and Th17), which is crucial for neuron survival and degeneration resistance (Goleij et al., 2025). In previous studies, peripheral blood IL-2 level decrease over time in PD patients whereas increase in MSA patients, indicating it may play different roles in the pathogenesis of different diseases (Csencsits-Smith et al., 2016) and recombinant IL-2 and IL-2/anti-IL-2 complexes seem to alleviate neuroinflammation and neurodegeneration, enhance Tregs function, and improve neuro-protection in PD mice (Li et al., 2025a; Goleij et al., 2025). These results suggest that IL-2 may play a role in the pathological process of neurodegenerative diseases by regulating neuroinflammation, but the underlying mechanisms still require further investigation. Additionally, changes of peripheral blood cytokines concentrations have been found to be associated with motor symptoms (e.g., IL-4 and IFN-γ), non-motor symptoms (e.g., IL-2 and IL-6), and regional gray matter volume (e.g., the IL-6 level are associated with the gray matter volume in the left precentral gyrus) in PD patients (Diaz et al., 2022; Kim et al., 2022; Chen et al., 2024). And changes of some cytokines (e.g., IL-2, IL-4, IL-9, and IL-15) are significantly faster in MSA patients compared to PD patients, which is similar to the conditions of their disease deterioration (Csencsits-Smith et al., 2016). These results suggest that the role of peripheral blood cytokines in neurodegenerative diseases may be more complex and extensive than we previously thought.

In previous studies, some alterations of peripheral immune system have been found to be stably related with neurodegeneration and might be related to increased risk of PD and MSA, such as lower lymphocyte counts, lower naive cells [e.g., naive CD4+/CD8+ T cells, and naive B cells], higher activated T cells and memory B cells, higher NK cells, and some evaluated cytokines (e.g., IL-1β, IL-6, and TGF-β1) (Zheng et al., 2024; Wang et al., 2023; Gong et al., 2023). These alterations are believed to be associated with adaptive immune system impairment, protective role of clearing α-syn and age-related immunosenescence, which are also associated with worse motor performance in neurodegenerative diseases (Malú Gámez et al., 2022; Lauritsen and Romero-Ramos, 2023). Additionally, prolonged levodopa treatment can also affect peripheral immune responses and reflect as lymphocyte subsets and cytokines changes in PD and MSA, such as CD19+ B cells decreasing, CD16+ lymphocyte increasing, and some cytokines decreasing (e.g., IL-4, IL-6, IL-10, TNF, and IFN-γ) (Ma et al., 2025). However, these results seem to be inconsistent across different studies (Furgiuele et al., 2023). As higher levodopa doses are usually in sync with PD/MSA progresses, it is difficult to conclude whether these alterations is related to disease progress or the treatment (Lauritsen and Romero-Ramos, 2023). Therefore, it is recommended to conduct more targeted large-scale clinical studies to clarify the relationship between neurodegenerative diseases and the peripheral immune system, especially the peripheral blood lymphocyte subsets and plasma cytokines.

## Limitations

Several limitations of this study should be noted. First, although our sample size met the basic research requirements, it was still relatively small, which might affect the statistical power. Large-scale multi-center and long-term cohort studies are required to validate our results. Second, no histopathological diagnosis was performed. Further tissue-level immunological analysis was needed to obtain more supportive evidence. We included only typical clinical cases to reduce the potential bias. Third, the cross-sectional design of this study determined that the results cannot establish causality or track dynamic changes of lymphocyte subsets/cytokines over time, thus further large-scale cohort studies are recommended. Fourth, we only evaluated alterations of peripheral blood lymphocyte subsets and plasma cytokines in MSA-P patients, whereas the cerebellar subtype of MSA (MSA-C) patients may have completely different peripheral immune profiles (Matsuse et al., 2020). Fifth, this study only used internal validation to assess the discriminatory performance of indicators, external and longitudinal validation is required to validate the results. Sixth, only flow cytometry was used to classify lymphocytes, without conducting TCR αβ*/*γδ phenotyping, cell status assessment, or function verification in this study. Further sub-classification of lymphocytes with multiple methods is recommended to facilitate deeper mechanistic interpretation. Seventh, factors such as sex, age, disease severity, and medication status might affect peripheral immune responses, and could be potential sources of residual confounding. Particularly, residual between-group differences still existed within different LEDD subgroups in sensitivity analysis, which could not be fully eliminated by stratification. It is recommended to control confounding variables via strategies such as single-sex recruitment, rigorous patient stratification, or only including drug-naive patients to obtain more reliable results.

## Conclusion

The count and percentage of CD4–CD8– T cells and IL-2 were significantly increased in MSA-P patients compared to PD patients and HCs; and the count and percentage of CD4–CD8– T cells and CD3+CD16+CD56+ NKT cells, and TNF-α were significantly increased in PD patients compared to HCs. When PD patients and MSA-P patients had similar clinical performance, CD4–CD8– T cells were significantly increased in MSA-P patients, and exhibited a satisfactory discriminatory capacity to differentiate MSA-P from PD, which had no statistically significant correlation with clinical indicators. Relevant conclusions needed to be verified through large-scale cohort studies.

## Data Availability

The raw data supporting the conclusions of this article will be made available by the authors, without undue reservation.

## References

[B1] Castellani G. Croese T. Ramos J. M. Schwartz M. (2023). Transforming the understanding of brain immunity. Science 380:eabo7649. doi: 10.1126/science.abo7649 37023203

[B2] Chen B. Zhou H. Liu X. Yang W. Luo Y. Zhu S. . (2024). Correlations of gray matter volume with peripheral cytokines in Parkinson's disease. Neurobiol. Dis. 201:106693. doi: 10.1016/j.nbd.2024.106693 39368669

[B3] Chen L. Mao L. Lu H. Liu P. (2023). Detecting ferroptosis and immune infiltration profiles in multiple system atrophy using postmortem brain tissue. Front. Neurosci. 17:1269996. doi: 10.3389/fnins.2023.1269996 38222105 PMC10784378

[B4] Csencsits-Smith K. Suescun J. Li K. Luo S. Bick D. L. Schiess M. (2016). Serum lymphocyte-associated cytokine concentrations change more rapidly over time in multiple system atrophy compared to Parkinson disease. Neuroimmunomodulation 23, 301–308. doi: 10.1159/000460297 28395279 PMC5491391

[B5] Diaz K. Kohut M. L. Russell D. W. Stegemöller E. L. (2022). Peripheral inflammatory cytokines and motor symptoms in persons with Parkinson's disease. Brain Behav. Immun. Health 21:100442. doi: 10.1016/j.bbih.2022.100442 PMC8927904 35308082

[B6] Fonseca M. Zanetti L. Fanourakis S. Sulzer D. L. Mazmanian S. K. (2026). The gut microbiome, systemic inflammation, and autoimmunity in Parkinson's disease. Lancet Neurol. 25, 103–114. doi: 10.1016/S1474-4422(25)00382-5 41389810

[B7] Furgiuele A. Pereira F. C. Martini S. Marino F. Cosentino M. (2023). Dopaminergic regulation of inflammation and immunity in Parkinson's disease: friend or foe? Clin. Transl. Immunol. 12:e1469. doi: 10.1002/cti2.1469 37781343 PMC10540835

[B8] Goleij P. Amini A. Tabari M. A. K. Hadipour M. Sanaye P. M. Alsharif K. F. . (2025). The role of interleukin (IL)-2 cytokine family in Parkinson's disease. Cytokine 191:156954. doi: 10.1016/j.cyto.2025.156954 40318236

[B9] Gong Z. Gao R. Ba L. Liu Y. Hou H. Zhang M. (2023). The peripheral immune traits changed in patients with multiple system atrophy. Brain Sci 13:205. doi: 10.3390/brainsci13020205 36831748 PMC9953988

[B10] Gregor K. W. Iva S. Luca V. Alessandra F. Giovanna C.-B. Klaus S. . (2022). The movement disorder society criteria for the diagnosis of multiple system atrophy. Mov Disord 37, 1131–1148. doi: 10.1002/mds.29005 35445419 PMC9321158

[B11] He Y. Peng K. Li R. Zhang Z. Pan L. Zhang T. . (2022). Changes of T lymphocyte subpopulations and their roles in predicting the risk of Parkinson's disease. J. Neurol. 269, 5368–5381. doi: 10.1007/s00415-022-11190-z 35608657 PMC9467943

[B12] Iyengar A. R. S. Dunkley P. R. Dickson P. W. (2025). Immunity in Parkinson's disease—the role of adaptive and auto-immune responses and gut-microbiome axis. J. Neuroimmunol. 409:578755. doi: 10.1016/j.jneuroim.2025.578755 40946701

[B13] Jensen M. P. Jacobs B. M. Dobson R. Bandres-Ciga S. Blauwendraat C. Schrag A. . (2021). Lower lymphocyte count is associated with increased risk of Parkinson's disease. Ann. Neurol. 89, 803–812. doi: 10.1002/ana.26034 33527442 PMC9012149

[B14] Kim R. Kim H.-J. Shin J. H. Lee C. Y. Jeon S. H. Jeon B. (2022). Serum inflammatory markers and progression of nonmotor symptoms in early Parkinson's disease. Mov. Disord. 37, 1535–1541. doi: 10.1002/mds.29056 35596676

[B15] Lauritsen J. Romero-Ramos M. (2023). The systemic immune response in Parkinson's disease: focus on the peripheral immune component. Trends Neurosci. 46, 863–878. doi: 10.1016/j.tins.2023.07.005 37598092

[B16] Li L. Gao W. Ren N. Chen L. (2025a). IL-2/anti-IL-2 complexes attenuates neuroinflammation and neurodegeneration in mice of experimental Parkinson's disease. Brain Res. Bull. 223:111273. doi: 10.1016/j.brainresbull.2025.111273 39999936

[B17] Li X. Guo D. Zou I. X. Zhao L. Yang N. Liu Y. (2025b). CD3+CD4–CD8– T cells: a new potential therapeutic target in treating autoimmune diseases. Front. Immunol. 16:1683418. doi: 10.3389/fimmu.2025.1683418 41080560 PMC12510931

[B18] Ma H. Wang Z. Yu M. Zhai Y. Yan J. (2025). Aberrations in peripheral B lymphocytes and B lymphocyte subsets levels in Parkinson disease: a systematic review. Front. Immunol.16:1526095. doi: 10.3389/fimmu.2025.1526095 40230858 PMC11994702

[B19] Malú Gámez T. Rebecca L W. Madelyn C H. Mary K H. Cody E K. Valerie J. (2022). Inflammation and immune dysfunction in Parkinson disease. Nat. Rev. Immunol. 22, 657–673. doi: 10.1038/s41577-022-00684-6 35246670 PMC8895080

[B20] Matsuse D. Yamasaki R. Maimaitijiang G. Yamaguchi H. Masaki K. Isobe N. . (2020). Early decrease in intermediate monocytes in peripheral blood is characteristic of multiple system atrophy-cerebellar type. J. Neuroimmunol. 349:577395. doi: 10.1016/j.jneuroim.2020.577395 32977251

[B21] Moon J.-S. Moutusy S. I. Zhang M. Ndayisaba A. Rodriguez D. Kodsi D. N. . (2026). Cytotoxic T cell recognition of α-synuclein drives pathogenic immune responses in multiple system atrophy. Proc. Natl. Acad. Sci. U. S. A. 123:e2537271123. doi: 10.1073/pnas.2537271123 41911451 PMC13056084

[B22] Moquin-Beaudry G. Andriamboavonjy L. Audet S. Hamilton L. K. Duquette A. Chouinard S. . (2025). Mapping the peripheral immune landscape of Parkinson's disease patients with single-cell sequencing. Brain 148, 2847–2860. doi: 10.1093/brain/awaf066 40417848 PMC12316021

[B23] Ndayisaba A. Halliday G. M. Khurana V. (2025). Multiple system atrophy: pathology, pathogenesis, and path forward. Annu. Rev. Pathol. 20, 245–273. doi: 10.1146/annurev-pathmechdis-051122-104528 39405585

[B24] Park T.-Y. Jeon J. Lee N. Kim J. Song B. Kim J.-H. . (2023). Co-transplantation of autologous Treg cells in a cell therapy for Parkinson's disease. Nature 619, 606–615. doi: 10.1038/s41586-023-06300-4 37438521 PMC12012854

[B25] Postuma R. B. Berg D. Stern M. Poewe W. Olanow C. W. Oertel W. . (2015). MDS clinical diagnostic criteria for Parkinson's disease. Mov. Disord. 30, 1591–1601. doi: 10.1002/mds.26424 26474316

[B26] Roodveldt C. Bernardino L. Oztop-Cakmak O. Dragic M. Fladmark K. E. Ertan S. . (2024). The immune system in Parkinson's disease: What we know so far. Brain 147, 3306–3324. doi: 10.1093/brain/awae177 38833182 PMC11449148

[B27] Velikkakam T. Gollob K. J. Dutra W. O. (2022). Double-negative T cells: setting the stage for disease control or progression. Immunology 165, 371–385. doi: 10.1111/imm.13441 34939192 PMC10626195

[B28] Wang M. Yan Z. Wang J. Yang Y. Deng Q. Han Y. . (2023). The characteristics and alteration of peripheral immune function in patients with multiple system atrophy. Front. Neurol. 14:1223076. doi: 10.3389/fneur.2023.1223076 37771450 PMC10525398

[B29] Wang P. Yao L. Luo M. Zhou W. Jin X. Xu Z. . (2021). Single-cell transcriptome and TCR profiling reveal activated and expanded T cell populations in Parkinson's disease. Cell. Discov. 7:52. doi: 10.1038/s41421-021-00280-3 34282123 PMC8289849

[B30] Whyte C. E. Singh K. Burton O. T. Aloulou M. Kouser L. Veiga R. V. . (2022). Context-dependent effects of IL-2 rewire immunity into distinct cellular circuits. J. Exp. Med. 219:e20212391. doi: 10.1084/jem.20212391 35699942 PMC9202720

[B31] Wu Z. Zheng Y. Sheng J. Han Y. Yang Y. Pan H. . (2022). CD3+CD4–CD8– (double-negative) T cells in inflammation, immune disorders and cancer. Front. Immunol. 13:816005. doi: 10.3389/fimmu.2022.816005 35222392 PMC8866817

[B32] Yan Z. Yang W. Wei H. Dean M. N. Standaert D. G. Cutter G. R. . (2021). Dysregulation of the adaptive immune system in patients with early-stage Parkinson disease. Neurol. Neuroimmunol. Neuroinflamm. 8:e1036. doi: 10.1212/NXI.0000000000001036 34301818 PMC8299515

[B33] Yuan X. Wan L. Chen Z. Long Z. Chen D. Liu P. . (2024). Peripheral inflammatory and immune landscape in multiple system atrophy: a cross-sectional study. Mov. Disord. 39, 391–399. doi: 10.1002/mds.29674 38155513

[B34] Zheng Y. Li Y. Cai H. Kou W. Yang C. Li S. . (2024). Alterations of peripheral lymphocyte subsets in isolated rapid eye movement sleep behavior disorder. Mov. Disord. 39, 1179–1189. doi: 10.1002/mds.29798 38529776

