# Supplementary material for: Alterations of peripheral blood lymphocyte subsets and plasma cytokines in Parkinson's disease and the parkinsonian subtype of multiple system atrophy
Source: Front Aging Neurosci. 2026 Oct 28;18:1990723. doi: 10.3389/fnagi.2026.1990723 (PMC13752209; doi:10.3389/fnagi.2026.1990723)
Supplement: Supplementary file 1 [file Table_1.docx]

**Supplementary-Table 1 Peripheral Blood Lymphocyte Subsets Analysis of PD Patients, MSA-P Patients, and HCs**

|  | | PD patients  (n=120) | MSA-P patients  (n=45) | HCs  (n=119) | Overall comparison | | | Between-group comparisons | | | | | |
| --- | --- | --- | --- | --- | --- | --- | --- | --- | --- | --- | --- | --- | --- |
|  |  |  |  |  | F | P | Partial η^2^ | PD vs HCs | | MSA-P vs HCs | | PD vs MSA-P | |
|  |  |  |  |  |  |  |  | p | FDR | p | FDR | p | FDR |
| Lymphocyte | count (*10^9^/L) | 1873.7±661.7 | 1887.3±545.4 | 1990.1±721.4 | 1.14 | 0.34 | 0.03 | 1.00 | 0.91 | 1.00 | 0.97 | 1.00 | 0.89 |
| CD3+ T cells | count (/μL) | 1348.3±502.7 | 1375.8±446.6 | 1454.5±619.6 | 1.25 | 0.27 | 0.04 | 1.00 | 1.00 | 1.00 | 0.89 | 1.00 | 0.89 |
|  | % | 72.3±10.5 | 72.8±7.1 | 72.3±8.2 | 1.62 | 0.12 | 0.05 | 1.00 | 0.97 | 1.00 | 0.89 | 1.00 | 0.91 |
| CD4+ T cells | count (/μL) | 778.1±315.7 | 836.8±274.4 | 890.0±350.2 | 2.35 | **0.02** | 0.06 | 1.00 | 0.75 | 1.00 | 0.78 | 1.00 | 0.97 |
|  | % | 41.8±9.6 | 44.4±7.2 | 44.6±8.4 | 2.84 | **<0.01** | 0.08 | 0.28 | 0.39 | 1.00 | 0.79 | 1.00 | 0.82 |
| CD8+ T cells | count (/μL) | 517.7±287.1 | 481.5±234.7 | 517.5±392.0 | 0.32 | 0.96 | 0.01 | 1.00 | 0.88 | 1.00 | 0.95 | 1.00 | 0.83 |
|  | % | 27.6±9.6 | 25.2±7.4 | 25.2±8.2 | 0.98 | 0.45 | 0.03 | 0.79 | 0.63 | 1.00 | 0.97 | 0.84 | 0.65 |
| CD4+CD8+ T cells | count (/μL) | 27.9±37.4 | 24.7±18.7 | 19.1±21.2 | 1.56 | 0.14 | 0.04 | 0.64 | 0.58 | 1.00 | 0.85 | 1.00 | 0.89 |
|  | % | 1.4±1.4 | 1.3±0.9 | 1.3±1.3 | 0.87 | 0.54 | 0.02 | 1.00 | 0.82 | 1.00 | 0.82 | 1.00 | 0.97 |
| CD4-CD8- T cells | count (/μL) | 134.0±86.8 | 182.0±82.8 | 61.6±48.0 | 15.09 | **<0.001** | 0.31 | **<0.001** | **<0.001** | **<0.001** | **<0.001** | **<0.01** | **0.02** |
|  | % | 7.2±4.0 | 9.7±3.6 | 4.4±3.3 | 10.80 | **<0.001** | 0.24 | **<0.001** | **<0.001** | **<0.001** | **<0.001** | **<0.001** | **<0.01** |
| CD19+ B cells | count (/μL) | 219.0±214.6 | 203.6±93.3 | 269.2±144.2 | 2.36 | **0.02** | 0.06 | 1.00 | 0.99 | 0.83 | 0.65 | 0.79 | 0.63 |
|  | % | 11.4±7.3 | 10.7±4.0 | 13.8±5.9 | 2.27 | **0.02** | 0.06 | 1.00 | 0.70 | 0.11 | 0.20 | 0.51 | 0.49 |
| CD16+CD56+ NK cells | count (/μL) | 287.6±246.1 | 287.0±142.4 | 252.3±163.2 | 1.69 | 0.10 | 0.05 | 0.66 | 0.59 | 0.30 | 0.39 | 1.00 | 0.82 |
|  | % | 15.2±9.9 | 15.4±6.5 | 13.0±7.0 | 3.96 | **<0.001** | 0.10 | 1.00 | 0.74 | 0.09 | 0.20 | 0.39 | 0.44 |
| CD3+CD16+CD56+ NKT cells | count (/μL) | 158.9±146.3 | 106.6±56.8 | 116.1±150.8 | 3.12 | **<0.01** | 0.08 | **<0.01** | **0.02** | 1.00 | 0.74 | 0.18 | 0.29 |
|  | % | 8.2±5.9 | 5.9±3.5 | 5.7±5.9 | 4.54 | **<0.001** | 0.12 | **<0.001** | **<0.01** | 0.93 | 0.67 | 0.11 | 0.20 |

NOTE: PD: Parkinson's disease, MSA-P: the Parkinsonian subtype of multiple system atrophy, HC: health control, NK cells: natural killer cells, NKT cells: natural killer T cells, FDR: false discovery rate.

MANCOVA (adjusts for groups, sex, age, MMSE, MoCA, HAMD, HAMA) was used to calculate overall comparison F, P and Partial η2. Post-hoc Bonferroni was used to calculate between-group comparison p, and the Benjamini-Hochberg method was used to adjust p-value to the FDR. Bold denotes p<0.05.

**Supplementary-Table 2 Main Effect Tests for MANCOVA Comparisons in Peripheral Blood Lymphocyte Subsets Analysis**

|  | | Overall comparison P | Groups  (PD/MSA-P/HCs) | Sex | Age | MMSE | MoCA | HAMD | HAMA |
| --- | --- | --- | --- | --- | --- | --- | --- | --- | --- |
| Lymphocyte | count (*10^9^/L) | 0.34 | 0.97 | 0.97 | 0.43 | 0.79 | 0.88 | 0.89 | 0.62 |
| CD3+ T cells | count (/μL) | 0.27 | 0.97 | 0.88 | 0.20 | 1.00 | 0.73 | 0.88 | 0.73 |
|  | % | 0.12 | 0.97 | 0.29 | 0.45 | 0.14 | 0.35 | 0.99 | 0.92 |
| CD4+ T cells | count (/μL) | **0.02** | 0.88 | 0.59 | **<0.05** | 0.86 | 1.00 | 0.89 | 0.70 |
|  | % | **<0.01** | 0.61 | **0.03** | 0.23 | 0.75 | 0.92 | 0.96 | 0.99 |
| CD8+ T cells | count (/μL) | 0.96 | 0.92 | 0.97 | 0.86 | 0.89 | 0.66 | 0.89 | 0.90 |
|  | % | 0.45 | 0.75 | 0.96 | 0.82 | 0.54 | 0.63 | 0.97 | 0.88 |
| CD4+CD8+ T cells | count (/μL) | 0.14 | 0.79 | 0.90 | 0.70 | 0.72 | 0.83 | 0.75 | 0.67 |
|  | % | 0.54 | 0.91 | 0.86 | 0.97 | 0.92 | 0.80 | 0.97 | 0.41 |
| CD4-CD8- T cells | count (/μL) | **<0.001** | **<0.001** | 0.60 | 0.70 | 0.74 | 0.79 | 0.97 | 0.92 |
|  | % | **<0.001** | **<0.001** | 0.24 | 0.83 | 0.85 | 0.88 | 0.89 | 0.94 |
| CD19+ B cells | count (/μL) | **0.02** | 0.81 | 0.75 | 0.31 | 0.85 | 0.78 | 0.89 | 0.44 |
|  | % | **0.02** | 0.41 | 0.99 | 0.30 | 0.99 | 0.88 | 0.99 | 0.67 |
| CD16+CD56+ NK cells | count (/μL) | 0.10 | 0.60 | 0.44 | 0.41 | 0.20 | 0.44 | 0.97 | 0.97 |
|  | % | **<0.001** | 0.39 | 0.20 | **0.02** | 0.12 | 0.20 | 0.99 | 0.88 |
| CD3+CD16+CD56+ NKT cells | count (/μL) | **<0.01** | **0.04** | 0.48 | 0.86 | 0.39 | **0.04** | 0.20 | 0.48 |
|  | % | **<0.001** | **0.02** | 0.44 | 0.72 | 0.12 | **0.03** | **0.03** | 0.11 |

NOTE: PD: Parkinson's disease, MSA-P: the Parkinsonian subtype of multiple system atrophy, HC: health control, NK cells: natural killer cells, NKT cells: natural killer T cells, FDR: false discovery rate.

The Benjamini-Hochberg method was used to adjust p-value to the FDR. Bold denotes p<0.05.

**Supplementary-Table 3 Plasma Cytokines Analysis Of PD Patients, MSA-P Patients, and HCs**

|  | PD patients  (n=120) | MSA-P patients  (n=45) | HCs  (n=119) | Overall comparison | | | Between-group comparison P | | | | | |
| --- | --- | --- | --- | --- | --- | --- | --- | --- | --- | --- | --- | --- |
|  |  |  |  | F | P | Partial η^2^ | PD vs HCs | | MSA-P vs HCs | | PD vs MSA-P | |
|  |  |  |  |  |  |  | p | FDR | p | FDR | p | FDR |
| IL-1β (pg/mL) | 2.8±1.7 | 3.4±2.4 | 2.6±2.6 | 1.18 | 0.31 | 0.03 | 0.73 | 0.61 | 0.31 | 0.40 | 1.00 | 0.82 |
| IL-2 (pg/mL) | 2.0±1.7 | 3.7±3.6 | 1.9±1.6 | 4.46 | **<0.001** | 0.11 | 1.00 | 0.99 | **<0.001** | **<0.01** | **<0.001** | **<0.01** |
| IL-4 (pg/mL) | 2.7±2.4 | 3.0±2.8 | 2.1±2.2 | 1.27 | 0.26 | 0.04 | 0.10 | 0.20 | 0.17 | 0.29 | 1.00 | 0.97 |
| IL-6 (pg/mL) | 4.1±3.0 | 5.7±3.4 | 4.0±2.7 | 1.96 | 0.05 | 0.05 | 1.00 | 0.88 | **<0.01** | **0.01** | **<0.01** | **0.02** |
| IL-10 (pg/mL) | 4.1±3.4 | 3.6±2.3 | 2.8±2.4 | 2.31 | **0.02** | 0.06 | **<0.01** | **0.03** | 0.29 | 0.39 | 1.00 | 0.75 |
| IL-12p70 (pg/mL) | 2.1±2.5 | 1.6±1.7 | 1.3±1.2 | 2.23 | **0.03** | 0.06 | **0.02** | 0.05 | 1.00 | 0.92 | 0.11 | 0.20 |
| IL-17 (pg/mL) | 5.4±5.8 | 5.4±4.8 | 3.8±4.3 | 1.94 | 0.05 | 0.05 | 0.45 | 0.45 | 0.74 | 0.62 | 1.00 | 0.99 |
| IFN-γ (pg/mL) | 4.7±4.6 | 4.8±3.8 | 3.9±2.6 | 0.76 | 0.64 | 0.02 | 0.42 | 0.44 | 0.27 | 0.39 | 1.00 | 0.88 |
| TNF-α (pg/mL) | 3.1±3.1 | 2.9±1.8 | 2.1±1.6 | 2.23 | **0.03** | 0.06 | **0.001** | **0.01** | 0.05 | 0.13 | 1.00 | 0.88 |

NOTE: PD: Parkinson's disease, MSA-P: the Parkinsonian subtype of multiple system atrophy, HC: health control, IL: interleukin, IFN: interferon, TNF: tumor necrosis factor, FDR: false discovery rate.

MANCOVA (adjusts for groups, sex, age, MMSE, MoCA, HAMD, HAMA) was used to calculate overall comparison F, P and Partial η2. Post-hoc Bonferroni was used to calculate between-group comparison p, and the Benjamini-Hochberg method was used to adjust p-value to the FDR. Bold denotes p<0.05.

**Supplementary-Table 4 Main Effect Tests for MANCOVA Comparisons in Plasma Cytokines Analysis**

|  | Overall comparison P | Groups  (PD/MSA-P/HCs) | Sex | Age | MMSE | MoCA | HAMD | HAMA |
| --- | --- | --- | --- | --- | --- | --- | --- | --- |
| IL-1β (pg/mL) | 0.31 | 0.61 | 0.95 | 0.39 | 0.37 | 0.44 | 0.92 | 0.86 |
| IL-2 (pg/mL) | **<0.001** | **<0.01** | 0.88 | 0.44 | 0.48 | 0.67 | 0.92 | 0.63 |
| IL-4 (pg/mL) | 0.26 | 0.29 | 0.88 | 0.64 | 0.60 | 0.78 | 0.45 | 0.56 |
| IL-6 (pg/mL) | 0.05 | **0.02** | 0.97 | 0.72 | 0.57 | 0.75 | 0.74 | 0.88 |
| IL-10 (pg/mL) | **0.02** | 0.09 | 0.45 | 0.71 | 0.82 | 0.88 | 0.43 | 0.67 |
| IL-12p70 (pg/mL) | **0.03** | 0.09 | 0.79 | 0.20 | 0.40 | 0.39 | 0.85 | 0.88 |
| IL-17 (pg/mL) | 0.05 | 0.67 | 0.92 | 0.89 | 0.12 | 0.06 | 0.92 | 0.89 |
| IFN-γ (pg/mL) | 0.64 | 0.51 | 0.82 | 0.89 | 0.67 | 0.92 | 0.92 | 0.99 |
| TNF-α (pg/mL) | **0.03** | **0.02** | 0.74 | 0.67 | 0.88 | 0.97 | 0.44 | 0.93 |

NOTE: PD: Parkinson's disease, MSA-P: the Parkinsonian subtype of multiple system atrophy, HC: health control, NK cells: natural killer cells, NKT cells: natural killer T cells, FDR: false discovery rate.

The Benjamini-Hochberg method was used to adjust p-value to the FDR. Bold denotes p<0.05.

**Supplementary-Table 5 Logistic Univariate and Multivariate Analysis of Peripheral Blood Lymphocyte Subsets Analysis and Plasma Cytokines Analysis between PD Patients and MSA-P Patients**

|  |  | Univariate analysis | | Multivariate analysis | |
| --- | --- | --- | --- | --- | --- |
|  |  | P | OR (95% CI) | P | OR (95% CI) |
| Lymphocyte | count | 0.90 | 1.00(1.00-1.00) | - | - |
| CD3+ T cells | count | 0.75 | 1.00(1.00-1.00) | - | - |
|  | % | 0.78 | 1.01(0.97-1.04) | - | - |
| CD4+ T cells | count | 0.27 | 1.00(1.00-1.00) | - | - |
|  | % | 0.10 | 1.04(0.99-1.08) | - | - |
| CD8+ T cells | count | 0.45 | 1.00(1.00-1.00) | - | - |
|  | % | 0.14 | 0.97(0.93-1.01) | - | - |
| CD4+CD8+ T cells | count | 0.59 | 1.00(0.99-1.01) | - | - |
|  | % | 0.68 | 0.94(0.71-1.25) | - | - |
| CD4-CD8- T cells | count | **<0.01** | 1.22(1.11-1.39) | **0.01** | 1.29(1.14-1.54) |
|  | % | **0.001** | 1.16(1.06-1.28) | **0.001** | 1.20(1.07-1.33) |
| CD19+ B cells | count | 0.65 | 1.00(1.00-1.00) | - | - |
|  | % | 0.55 | 0.98(0.93-1.04) | - | - |
| CD16+CD56+ NK cells | count | 0.99 | 1.00(1.00-1.00) | - | - |
|  | % | 0.93 | 1.00(0.97-1.04) | - | - |
| CD3+CD16+CD56+ NKT cells | count | **0.03** | 0.99(0.99-1.00) | **0.02** | 0.99(0.98-1.00) |
|  | % | **0.01** | 0.88(0.79-0.98) | **0.01** | 0.83(0.72-0.96) |
| IL-1β |  | 0.10 | 1.15(0.97-1.37) | - | - |
| IL-2 |  | **<0.001** | 1.38(1.15-1.65) | **<0.01** | 1.41(1.18-1.71) |
| IL-4 |  | 0.56 | 1.04(0.91-1.19) | - | - |
| IL-6 |  | **<0.01** | 1.17(1.05-1.30) | **<0.01** | 1.24(1.07-1.43) |
| IL-10 |  | 0.33 | 0.94(0.84-1.06) | - | - |
| IL-12p70 |  | 0.22 | 0.89(0.73-1.08) | - | - |
| IL-17 |  | 1.00 | 1.00(0.94-1.06) | - | - |
| IFN-γ |  | 0.88 | 1.01(0.93-1.09) | - | - |
| TNF-α |  | 0.65 | 0.97(0.86-1.10) | - | - |

NOTE: PD: Parkinson's disease, MSA-P: the Parkinsonian subtype of multiple system atrophy, HC: health control, NK cells: natural killer cells, NKT cells: natural killer T cells, IL: interleukin, IFN: interferon, TNF: tumor necrosis factor, OR: odds ratio, CI: confidence interval.

Binary logistics regression analysis (adjusted for sex, age, symptom duration, UPDRS-III score, MMSE, MoCA, LEDD, HAMD, and HAMA) was used.

Bold denotes p<0.05.

**Supplementary-Table 6 Stability of the Multivariable Logistic Regression Models**

|  | CD4-CD8- T cells | | CD3+CD16+CD56+ NKT cells | | IL-2 | IL-6 |
| --- | --- | --- | --- | --- | --- | --- |
|  | count | % | count | % |  |  |
| **Variance Inflation Factor Analysis** | | | | | |  |
| VIF | 1.15 | 1.19 | 6.04 | 2.04 | 1.18 | 4.37 |
| **Bootstrap resampling (1000 replicates)** | | | | | |  |
| OR (95% CI) | 1.27(1.12-1.58) | 1.19(1.05-1.36) | 0.99(0.97-1.01) | 0.82(0.69-0.98) | 1.40(1.15-1.77) | 1.22(1.04-1.46) |

NOTE: VIF: variance inflation factor, OR: odds ratio, CI: confidence interval.

**Supplementary-Table 7 Demographic and Clinical Features of PD Patients and MSA-P Patients in Sensitivity Analysis**

|  |  | PD patients | | MSA-P patients | | P |
| --- | --- | --- | --- | --- | --- | --- |
|  |  | n | Mean ± SD | n | Mean ± SD |  |
| Sex | male/female | 51/69 | - | 18/27 | - | 0.77 |
| UPDRS-III score | >20 | 56 | 27.25±4.66418 | 22 | 26.6818±5.33083 | 0.64 |
|  | ≤20 | 64 | 12.75±4.40779 | 23 | 14.8696±4.89252 | 0.06 |
| LEDD (mg/day) | >275 | 72 | 543.64±232.31 | 13 | 485.10±175.62 | 0.39 |
|  | ≤275 | 48 | 102.60±98.25 | 32 | 34.38±77.71 | **<0.001** |

NOTE: PD: Parkinson's disease, MSA-P: the Parkinsonian subtype of multiple system atrophy, SD:standard deviation, UPDRS-III: Unified Parkinson's Disease Rating Scale-part III, LEDD: levodopa equivalent daily dose.

Bold denotes p<0.05.

**Supplementary-Table 8 Covariate Balance Before and After Inverse Probability Weighting**

| Covariates | r before weighting | r after weighting |
| --- | --- | --- |
| sex | -0.09 | -0.08 |
| age | -0.03 | 0.02 |
| symptom duration | -0.03 | -0.03 |
| UPDRS-III score | -0.07 | -0.06 |
| MMSE | 0.09 | 0.11 |
| MoCA | 0.09 | 0.10 |
| LEDD | -0.05 | -0.04 |
| HAMD | -0.07 | -0.05 |
| HAMA | -0.09 | -0.08 |

NOTE: r: Pearson correlation coefficient between covariates and the count of peripheral blood CD4-CD8- T cells; adequate balance defined as |r| <0.1.

**Supplementary-Figure 1 Representative Flow Cytometry Plots of Peripheral Blood Lymphocyte Subsets Analysis for MSA-P Patients and HCs**

**(a)** representative flow cytometry plots from a MSA-P patient

**(b)** representative flow cytometry plots from a healthy control

Note: A-B: identify lymphocytes from white blood cells (CD45+); C: identify T cells (CD45+CD3+) from lymphocytes; D: identify CD4+ T cells (CD45+CD3+CD4+); E: identify CD8+ T cells (CD45+CD3+CD8+); F: identify B cells (CD45+CD3-CD19+); G: identify NK cells (CD45+CD3-CD16+CD56+) and NKT cells (CD45+CD3+CD16+CD56+); H: identify CD4+CD8+ T cells (CD45+CD3+CD4+CD8+) and CD4-CD8- T cells (CD45+CD3+CD4-CD8-).

A-C: CD45+CD3+ lymphocytes were set as blue, and CD45+CD3- lymphocytes were set as red; D-G: red/blue was set to distinguish CD3-/CD3+.

When calculating percentages of each lymphocyte subset, the count of total lymphocytes was set as the denominator.

(a) and (b) showed flow cytometry plots from a MSA-P patient and a healthy control respectively.
